# Off-label prescribing for allergic diseases in children

**DOI:** 10.1186/1939-4551-7-4

**Published:** 2014-02-14

**Authors:** Diana Silva, Ignacio Ansotegui, Mário Morais-Almeida

**Affiliations:** 1 Immunoallergology Department, Centro Hospitalar de São João, Alameda Prof. Hernâni Monteiro, 4200-309 Porto, Portugal; 2 Immunoallergology Department, CUF Descobertas Hospital, R. Mário Botas, 1998-018 Lisboa, Portugal; 3 Department of Allergy and Immunology, Hospital Quirón Bizkaia, Carretera de Leioa-Unbe, 33 Bis., 48950 Erandio, Spain; 4 Center for Research in Health Technologies and Information Systems, University of Porto, Porto, Portugal

**Keywords:** Child, Preschool child, Off-label use, Unlicensed, Asthma, Urticaria, Atopic dermatitis, Rhinitis, Anti-asthmatic agents, Drugs

## Abstract

The majority of drugs prescribed have not been tested in children and safety and efficacy of children’s medicines are frequently supported by low quality of evidence. Therefore, a large percentage of prescriptions for children in the clinical daily practice are used off label. Despite the several recent legislation and regulatory efforts performed worldwide, they have not been successful in increasing availability of medicines adapted to children. Moreover, if we consider that 30% of the prescribed drugs for children are for the respiratory field and only 4% of new investigation projects for children research were proposed to access drugs for respiratory and allergy treatment, there is a clear imbalance of the children needs in this therapeutic area. This narrative review aimed to describe and discuss the off-label use of medicines in the treatment and control of respiratory and allergic diseases in children. It was recognized that a large percentage of prescriptions performed for allergy treatment in daily clinical practice are off label. The clinicians struggle on a daily basis with the responsibility to balance risk-benefits of an off-label prescription while involving the patients and their families in this decision. It is crucial to increase awareness of this reality not only for the clinician, but also to the global organizations and competent authorities. New measures for surveillance of off-label use should be established, namely through population databases implementation. There is a need for new proposal to correct the inconsistency between the priorities for pediatric drug research, frequently dependent on commercial motivations, in order to comply to the true needs of the children, especially on the respiratory and allergy fields.

## Introduction

The majority of drugs prescribed have not been tested in children and safety and efficacy of children’s medicines are frequently supported by low quality of evidence [1]. In Europe the percentage of authorized medicines for children is 33.3% [2]. This is explained by the lack of clinical research in this population, caused by ethical, scientific and technical issues, but also commercial priorities [3,4]. Therefore, most of the therapies prescribed to children are on an off-label or unlicensed basis [1].

Global legislation and regulatory efforts have been done to overpass these limitations aiming to produce proper research in the pediatric population, promoted by an International Conference on Harmonization (ICH) guidelines for clinical investigation of medicinal products in the pediatric population [5]. Since 1997, the Food and Drug Administration (FDA) in the United States of America (US) produced several regulation/legislation initiatives (Pediatric Rule Regulation, 1998; Best Pharmaceutical for Children Act, 2002 and Pediatric Research Equity Act, 2003) [6,7]. In Europe (EU), followed by US experiences, new regulations were implemented since January 2007 [8]. In both continents the measures taken enclosed financial incentives to the industry, the addition of 6 months extra patent protection and an additional 2 years market exclusivity for orphan medicines [1,3]. Furthermore, World Health Organization (WHO) adopted in 2007 the WHA60.20 Resolution “Better Medicines for Children” to undertake activities in the interest of improving pediatric medicines research, regulation and rational [4]. One of the most important was the establishment of the Model List of Essential Medicines for Children, now in its 4th version [9]. However, major discrepancies between drug prescription patterns in children and the drugs granted pediatric exclusivity still exists. Looking back to the last 5 years of the Pediatric Regulation from the European Medical Agency (EMA) [Regulation(EC) N°1901/2006], 600 pediatric investigation plans (PIP) were performed, of those 453 referred to not yet authorized drugs, while the remaining related to new indications. However, no specific therapeutic area was addressed more than the other, and as far as pneumology and allergology are considered, they only accounted for 4% of PIP [10]. At the same time, 30% of the prescribed drugs for children are for the respiratory system. This suggests that pediatric studies still do not address the real need in pediatric drug development despite an overall increase of medicines now available for children. Most of the drugs available on the market, specially those considered for the treatment of allergic diseases, are still not specifically tested in children, particularly in the younger ones. The aim of this review is to describe and discuss the current off-label use of medicines in the treatment and control of allergic diseases in children.

## Review

### Definitions and concepts

For approval of a new medicine, the manufacturer is required to provide the relevant national medicines regulatory authority specific information about its quality, safety and efficacy. When successful, the new medicine/formulation is approved and a Marketing Authorization is issued along with the Summaries of Product Characteristics (SPC) [11]. However, the use of drugs outside their authorized SPC is not the concern of the authorities and is the sole responsibility of the prescriber [12].

Off-label use (unlabelled or unapproved) refers to prescription and/or administration of a drug outside the terms of the marketing authorization, in a way not detailed in the SPC. An unlicensed (unregistered) use is described as a formulation or dosage that has not been approved in the country in which it is prescribed or administered [11,13,14]. However, in the literature, the exact definition varied between authors through time. In a recent systematic review, different off-label types of use were found, some considered dose, frequency and route of administration, while others only contra-indications or age range [11]. In an effort to produce a common definition for future research and regulatory purposes, Neubert *et al.* through a systematic review of the literature and a Delphi survey with 34 experts from different areas, provided common definitions for off-label drug use in children [14]. “*Pediatric off-label use*” included all pediatric uses of a marketed drug not detailed in the SPC, namely: therapeutic indication; therapeutic indication for use in subsets (like age groups); appropriate strength (dosage by age); pharmaceutical form and route of administration [14]. For the purposes of this review this off-label definition was considered.

### Trends of off-label prescription in children

To ascertain the trends of off-label use in children, especially in respiratory and allergic diseases, a systematic search of the literature was performed in Pubmed-Medline in July 2013 using the terms associations, *“(off-label OR unlicensed OR unapproved OR unregistered)”* AND *“children”*. The studies approaching the general prevalence of off-label use that reported allergic and/or respiratory diseases data are described in Table 1. The percentage of off-label use varied widely between studies, ranging from 3 to 51% of prescriptions, and reaching a prevalence of 78%, when considering patients that received at least one off-label medicine. This variability can be explained by the different settings (countries), age range and population sample (outpatient, inpatient, population databases from pharmacies or from medical prescription records). Sturkenbbom *et al.*, compared three different countries prescription patterns (Italy, United Kingdom and Netherland) that, despite being quite similar, off-label prescriptions percentages differed, which could be explained by different pediatric authorization status of the drugs in these countries [15]. A systematic review assessing off-label prescription in children found it to be common in all settings, but higher rates were seen for neonatal versus pediatric wards and for hospital versus community settings [16]. Therefore, off-label prescription should be assessed carefully and adapted to each reality and population.

**Table 1:** Summary of the studies reporting off-label medicines use and specifying respiratory off-label use

								**Off-label%**	
**Reference**	**Country**	**Setting**	Study design*	**Age**	**No patients**	**No Prescriptions**	**Off-label type**	**Total**	**Resp.**
Knopf, 2013 [17]	Germany	Population based sample (K*iGGS*)	Prospective; drug-use assessed by survey	0 to 17 years	8899	12667	Age, dose, indication	30	37
Morais-Almeida, 2013 [12]	Portugal	Allergy outpatient clinic	Retrospective; clinical files analysis in 2012	0 to 6 years	500	1224	Age, dose, indication	35	77
Ribeiro, 2013 [18]	Portugal	ED; University Hospital	Retrospective; random sample of children attending to the ER for 9 months in 2010	0 to 17 years	700	724	Age, dose, indication, route	32	28
Ballard, 2012 [19]	Australia	Pediatric general ward, acute-care university Hospital	Retrospective; two groups of 150 consecutive pediatric patients admitted in July 2009 and Jan. 2010	1 day to 11 years	300	887	Age, dose, indication, route	32	11
Kimland, 2012 [20]	Sweden	34 Pediatric; 7 non-Pediatric Hospitals	Prospective; data collection of all prescriptions, in two separate 48 hour periods (May and October 2008)	0 to 18 years	2947	11 294	Age, dose, indication, route	34	11
Palcevski, 2012 [21]	Croatia	Pediatric Ward; University Hospital	Prospective; clinical files analysis on a pre-determined day of each month during 12 months (May 2010 to April 2011)	0 to 19 years	531	1643	Age, indication, route	13.3	5.1
Olsson, 2011 [22]	Sweden	Population based sample (Swedish Prescribed Drug Register)	Retrospective; analysis of all outpatient prescriptions performed in 2007	0 to 18 years	--	2.19 million	Age, dose, indication	13.5	3.1
Phan, 2010 [23]	US	ED of a tertiary-care children’s Hospital	Retrospective; all medical records admissions analysis from January to May 2007	0 to 18 years	2191	6675	Age, dose, Indication, route	25.6	31.8
Morales-Carpi, 2010 [24]	Spain	Outpatient prescriptions	Prospective; analysis of all prescriptions performed prior to the ED visit collected from June2005 to August 2006 (14 months)	0 to 14 years	336	667	Indication, dose, frequency and route	50.7	31.4
		Join outpatient prescriptions to ER random sample; University Hospital							
MuhlBauer 2009 [25]	Germany	German statutory health insurance provider	Retrospective; analysis of all prescriptions performed during 2002	0 to 16 years	--	1.5 million	Age, indication	3.2	7
Bazzano, 2009 [26]	US	National Ambulatory Medical Care Surveys (NAMCS)	Retrospective; representative sample of outpatient visits from 2001 to 2004	0 to 18 years	312 million	484 million	Age, indication	62^#^	70^#^
Jain, 2008 [27]	India	Tertiary care central Hospital	Prospective study, prescription survey applied to a consecutive sample of children admitted to the ward from May to July 2006	1 month to 12 years	600	2064	Age, dose, frequency, indication	51	53
Hsien, 2008 [28]	Germany	Pediatric ward in tertiary care Hospital	Prospective study of all patient files between January and June 2006	0 to 18 years	417	1812	Age, dose, indication	31	30
Shah, 2007 [29]	US	31 tertiary care pediatric hospitals (*PHIS database*)	Retrospective study of all children discharged from the Hospital during 2004	0 to 17 years	355409	---	Age, indication	78.7^#^	11.2^#^
Ufer, 2004 [30]	Sweden	Population based sample (*Statistics Sweden and the National Corporation of Swedish Pharmacists*)	Retrospective study of all drug register present in the database in 2000	0 to 15 years	--	2,8million	Age, dose, indication, formulation, route	20.7	8.6
Schirm, 2003 [31]	Netherland	Pharmacies dispensing records in northern Netherland (*Interaction database*)	Retrospective study of all drugs dispensing records in the Interaction database in 2000	0-16 years	18493	66222	Age	20.6	15.1
Pandolfini, 2002 [32]	Italy	Nine general pediatric hospitals wards	Prospective; analysis of all prescriptions performed to children in 12 week period	1 month to 14 years	1461	4255	Dose, route, indication and duration	60	33
McIntyre, 2000 [33]	England	Suburban general practice clinic	Retrospective; study of all prescriptions performed in 1998	0 to 12 years	1175	3347	Age, dose, route	10.5	28

*All studies had a cross-sectional study design; ED- Pediatric Emergency department; #- off-label percentages is reported to visits or patients that received at least 1 off-label-drug; KiGGS- German Health Interview and Examination Survey for Children and Adolescents; PHIS- Pediatric Health Information System.

Frequency of drug prescription increases with age; however the number of off-label medicines use decreases. The highest proportion of off-label prescription in children occurs in the first two years of life [18,20,22,24-26,29,31,34-36]. In an outpatient setting in the US, the adjusted probability of receiving at least one off-label prescription in a medical visit was 59% in children’s aged 6 to 12 years, increasing to 65% from 2 to 6 year of age, to 67% if they had 1 to 2 years of age and 74% if less than 1 year (p < 0.001) [26]. Furthermore, probability could increase by 26 to 39% if they received more than one drug (p < 0.001) [26]. Nevertheless, this is not consistent in all studies. In a recent outpatient population based sample analysis in Germany there was a predominance of off-label medication use from 3 to 13 years of age [17]. The main reason for this difference can be related with the study population, mainly composed by healthy children. When considering children that resort to health care *versus* those that are hospitalized results differ. In a study addressing children admitted to different pediatric wards the odds of being prescribed an off label drug almost doubled in children with less than 1 year of age (OR 1.80; 95% CI 1.03–3.59, adjusted to age, gender, number of medications prescribed and type of ward) [37]. At the same time, several other factors besides age interfere with the use of off label medicines. The other most commonly encountered reason for off-label prescribing was dosage, that both includes under-dosing and over-dosing [16,17,19,20,27,30,33,35],[38,39]. This is expected due to the frequent dose adjustments needed to be performed in children. Other frequently reported reasons were unapproved therapeutic indication [18,22], followed by inappropriate age [17-19,24,38], frequency of drug use and, as less frequently reported, route of administration [19,32] and type of formulation [21]. The total absence of pediatric information in the SPC is also a common problem in off label prevalence studies. Inconsistent information between SPC was noted, namely for drugs with the same active compound but from different companies [20,40].

### Drug related problems and off-label drug use in children

Off-label prescribing is not illegal, not necessarily wrong, and is contemplated in several pediatric guidelines, but remarkably, no reference is made that some drugs are being recommended in an unlicensed or off-label use basis [41]. Indeed, quality of drug therapies is not necessarily related to drug license status [16]. However this has several clinical, ethical and safety issues and there is no explicit guide to help clinicians assess the appropriateness of off-label prescribing [21]. Often it is necessary to use medicine in an off-label basis, but this should be appraised according to clinical indications, therapeutic alternatives and risk-benefit analysis, and it is required to obtain informed consent from the patient or guardian [12]. Repeatedly a question is posed in the literature [11] and clinicians minds: “*Is off-label use more likely to be implicated in an Adverse Drug Reaction?”*

A recent review that accessed the relationship between off-label and unlicensed medicine use and adverse drug reactions (ADR) in children concluded that good quality of evidence is lacking to answer this question; different methodologies are used and definitions of off-label and unlicensed are not consensual between studies [11]. However, results of previous studies have indicated that there might be an association between off-label use and ADR risk [11]. In all ADRs reported over a decade in Danish children, one-fifth was associated with off-label prescriptions [42].

Evidence has been contradictory and varies widely. In studies with prospective design, incidence of ADR in off-label drugs ranged from 2 to 39% [11]. Santos *et al.* reported that in an inpatient population, off-label drug use was significantly associated with ADRs (relative risk 2.44; 95% CI 2.12, 2.89) [39]. In another inpatient sample, Neubert *et al.* reported a higher prevalence of ADR with off-label use compared with licensed ones (6.1 *versus* 5.6%). On the other hand, evaluating an outpatient setting showed a frequency of ADR 2-fold higher among licensed medication than in off-label, though the overall frequency of ADR was low (<1%) [23].

Respiratory diseases treatments have been reported in several associations with adverse reactions and off-label prescription. In a retrospective analysis of all ADR reported from the Swedish Drug Information System in 2000, medications used for asthma treatment were the most frequently associated with adverse reactions. Of those, 31% were being used off-label [30]. In another study regarding a pharmacovigilance prospective survey in France, exposure to drugs of “Respiratory System” in a multivariate analysis was associated with a decreased risk on ADR (0.20; 95% CI 0.07, 0.60). This study also related the off-label use of a drug due to a different indication with an increased risk of ADR, particularly in infants (3.94; 95% CI 1.12, 13.84) [43]. Several factors interfere in this unknown relationship of off-label medicines and ADR, as age, type of drug, disease and previous evidence of that medication use. Any decision about off-label prescription should weight risks and benefits and has to be based on value judgments that must involve parents or guardians in the decision [44].

### Off-label prescription for asthma treatment in children

Considering the global trends of outpatient prescription in children, allergy and asthma medicines are on the top of the most dispensed drugs [45]. If only respiratory medication is considered, asthma therapies are the most frequently prescribed (40.7% of all prescriptions) [46]. Therefore, knowledge of the authorized drugs for asthma is essential for adequate patient care. The most commonly used drugs for treating asthma are presented in Table 2, accordingly to the authorized age and maximum allowed dose limits.

**Table 2:** Drugs used for treatment of asthma in children and authorizations for their use according to age, dose and indication

**Category**	**Drug**	**Indication**	**Age lower limit**		**Maximum allowed dose**	
			Europe*	**USA**^**#**^	Europe*	**US**^**#**^
**Inhaled corticosteroids**	Budesonide (DPI; MDI)	Asthma prophylactic treatment	2 years	6 years	400 μg/day – 2 to 6 years	800 μg/day
					800 μg/day – 6 to 18 years	
	Budesonide (INH)		6 months	12 months	2000 μg/day	500 μg/day
	Fluticasone (DPI; MDI)		12 months	4 years	200 μg/day – 1 to 4 years;	200 μg/day (DPI) or 176 μg/day (MDI)– 4 to 11 years;
					400 μg/day – 4 to 16 years	2000 μg/day (DPI) or 1760 μg/day (MDI) ≥ 12 years
					2000 μg/day > 16 years	
	Mometasone furoate (DPI)		12 years	4 years	800 μg/day	110 μg/day – 4 to 11 years;
						880 μg/day ≥ 12 years
	Beclomethasone Dipropionate		4 years	5 years	400 μg/day – 4 to 12 years;	160 μg/day – 5 to 11 years;
					2000 μg/day ≥ 12 years	640 μg/day ≥ 12 years
**Oral anti-leukotrienes**	Montelukast		6 months	6 months	4 mg/day – 6 months to 5 years;	
					5 mg/day – 6 to 14 years	
					10 mg/day ≥15 years	
**Short-acting β2 agonists**	Salbutamol or Albuterol	Asthma reliever treatment	None	4 years (MDI)	800 μg/day <12 years (MDI)	1080 μg/day (MDI)
				2 years(INH)	10 mg/day < 12 years (INH)	5 mg/day- 2 to 12 years (INH)
					20 mg/day ≥ 12 years(INH)	10 mg/day ≥ 12 years (INH)
	Terbutaline (DPI)		3 years	---	4 mg/day – 3 to 12 years	---
					6 mg/day ≥ 12 years	
**Long-acting β2 agonists**	Salmeterol	Asthma prophylactic treatment	4 years (MDI; DPI)	4 years (DPI)	100 μg/day	
	Formoterol (DPI)		6 years	5 years	24 μg/day – 5 to 12 years	24 μg/day
					48 μg/day ≥ 12 years	
**Combination long-acting β2 agonists with inhaled corticosteroids**	Budesonide/Formoterol (DPI)		6 years	12 years	320/18 μg/day – 6 to 12 years	640/18 μg/day
					640/18 μg/day ≥ 12 years	
	Fluticasone/Salmeterol		4 years(DPI)	4 years (DPI)	200/100 μg/day (DPI; MDI)- 4 to 11years	200/100 μg/day (DPI)- 4 to 11years
			4years(MDI)	12 years (MDI)	1000 μg/100day (DPI; MDI)- ≥12 years	1000 μg/100day (DPI)- ≥12 years
						460/42 μg/day (MDI)

DPI- dry powder inhaler; MDI- Metered dose inhaler; INH- inhalation suspension *Data obtained in the Summaries of Product Characteristics (SPC) of one European country (Portugal, *Infarmed*[47]), except for mometasone furoate obtained from the UK SPC ^**#**^Data obtained in the FDA approved Drugs Database [48].

Comparison between an European country (regulated by EMA and the national authority) and the United States of America (regulated by FDA).

Asthma management guideline recommendations, namely Global Initiative for Asthma (GINA) or Expert Panel Report 3: Guidelines for the Diagnosis and Management of Asthma (EPR-3) [49,50] are widely followed by physicians, and both provide recommendations for all age groups; however, evidence supporting recommendations for preschool children is limited. The mainstay treatment for asthma are inhaled corticosteroids (ICS), but guidelines often do not provide specific recommendations for upper doses limits specially in children [50]. In this age group, namely in preschool children, inhaled corticosteroids are also the most recommended for long-term asthma treatment, mainly based in the previous experience in adults and in older children, though they advise that dose-responses are not well studied [49-51] and indeed “children are not a small size adult”. Asthma treatment poses several challenges in very young children; often an overlap between recurrent wheezing and asthma phenotypes occurs, making diagnosis and therapeutic decisions controversial [52,53]. Moreover, some therapeutic options are not deprived of side effects [49,52].

In children, inhaler type and child’s ability to use it correctly also interferes with the treatment. Beclomethasone, budesonide and fluticasone are available as either metered dose inhaler (MDI) or dry powder inhaler (DPI). Preschool children are not able to cooperate with the proper inhalation technique demanded by DPI, therefore these devices are not licensed for this population [52]. Furthermore, some new drugs like mometasone and ciclesonide are still not approved under 12 years [52].

In long-term treatment, if control is not achieved, other treatment associations can be considered, but their efficacy and safety are also not established in some cases. Long acting β2 agonists (LABA) are endorsed in the EPR-3 as one option for step-up therapy for persistent asthma in association to inhaled corticosteroids. Though they advise that these drugs aren’t adequately studied in children with less than 4 years of age, they are recommended as an add-on in the upper steps of the stepwise approach [50]. On the other hand, GINA guidelines specifies asthma therapy for children under 5 years of age, where LABA aren’t approved, indicating oral anti-leukotriene’s as an option [54,55]. Guidelines are recommendations on the appropriate management, diagnosis and treatment, but they differ between each other and vary widely between countries, however they do not replace clinician’s knowledge and skills. Several studies assessed the pediatric use of asthma drugs in different countries through cohort and cross-sectional studies [46,56,57]. TEDDY study is a 6 years retrospective analysis of outpatient medical records concerning pediatric asthma that combined databases from Netherland, Italy and United Kingdom and described the use of asthma drugs to be more frequent in children less than two years of age [56] to whom drug authorizations are scarce (Table 2). As expected, asthma treatment in children under 2 years present the highest prevalence of off-label use [12,46,56,57]. The most frequent drugs used off label are the short acting β2 agonist (SABA) salbutamol, ranging from 24 to 45% [56,57], and inhaled corticosteroids, from 26 to 80% [12,56,57]. Fixed combination of ICS and LABA were also often prescribed [56]. When considering types of off-label use, salbutamol and ICS were the most frequently reported due to age limits (19%), and salmeterol-fluticasone association due to inadequate indication [33,57]. Other studies report off-label use of these drugs due to higher than recommended doses; this could be explained mainly owing to inconsistencies found between the SPC and country guideline recommendations [19,35]. Many children with asthma are not managed in accordance with the set guidelines, as they vary widely in the literature and are not consistent with the SPC, leaving physicians to prescribe off-label. Most recognize it and believe that off-label prescription is appropriate, however they have efficacy and safety concerns. Moreover in a recent study only one third of the physicians self-reported that children’s guardians/parents were informed of off-label treatment use [58].

### Off-label use for rhinitis treatment in children

Allergic rhinitis is the most prevalent chronic allergic disease in children [59]. Oral second-generation anti-histamines and intranasal corticosteroids are considered the first line treatments [59-61]. In Table 3 are described some examples of the most often used drugs for allergic rhinitis, accordingly to the age limit and maximum allowed dose. The majority of intranasal corticosteroids and some of the anti-histamines lack pediatric approval and this is recognized by the guidelines [59,61]. These drugs are recommended for children by extrapolation from pharmacological and clinical data in adults. However, the absorption, distribution and metabolism in children diverge from adults and age-related differences in children exist in their ability to metabolize, absorb, excrete and transform medications, therefore efficacy and safety might be affected [59,62]. Although nasal corticosteroids can be associated with some side effects, including bone mineral density loss, adrenal suppression and growth retardation, these were only reported in one study using beclomethasone [60,62]. Therefore, only the lowest possible dose for symptoms control is favored [61]. Intranasal corticosteroid use before age of 2 is considered off-label and only mometasone is authorized in less than 4 years of age in the US (Table 3). As for anti-histamines, accordingly to the most recent guideline update, first generation drugs should not be used for rhinitis in children due to their side effects [63]. However, the most frequently available anti-histamines over the counter in the US are from first generation. This raised public health concerns about their use in children and, in the US, campaigns have been conducted to advise for safety concerns and recommend against their use under the age of two [64]. If considering an outpatient setting, the majority of off-label prescriptions were from pediatricians (54.4%), but a large number, 34.3%, were self-medications [24]. Despite their widely use only few studies have been performed to assess the magnitude of off-label drug in children for rhinitis.

**Table 3:** Drugs used for treatment of allergic rhinitis, urticaria and atopic eczema in children and their authorizations for their use according to age, dose and indication

**Category**	**Drug**	**Indication**	**Age lower limit**		**Maximum allowed dose**	
			**Europe**	**USA**	Europe*	**US**^**#**^
**Nasal inhaled corticosteroids**	Budesonide	Allergic Rhinitis	6 years	6 years	400 μg/day	400 μg/day
	Fluticasone furoate		4 years	4 years	50 μg/day – 4 to 12 years	200 μg/day
					200 μg/day ≥ 12 years	
	Mometasone		6 years	2 years	100 μg/day	100 μg/day -2 to 11 years;
						200 μg/day ≥ 12 years
**Oral antihistamines**	Cetirizine	Allergic rhinitis; Urticaria	2 years	6 months	5 mg/day – 2 to 6 years;	2.5 mg/day- 6 months to 1year
					10 mg/day > 6 years	5 mg/day- >1 year to 5 years
						10 mg ≥ 6 years
	Levocetirizine		2 years	6 months	2.5 mg/day – 2 to 6 years;	1.25 mg 6 months to 5 years
					5 mg/day – older than 6 years	2.5 mg- 6 years to 11 years
						5 mg ≥ 12 years
	Loratadine		2 years	2 years	5 mg/day – 2 to 6 years;	
					10 mg/day > 6 years	
	Desloratadine		12 months	6 months	1.25 mg/day – 1 to 5 years;	1 mg – 6 to 11 months
					2.5 mg/day – 6 to 12 years;	1.25 mg/day – 1 to 5 years;
					5 mg/day ≥ 12 years;	2.5 mg/day – 6 to 11 years;
						5 mg/day ≥ 12 years
	Fexofenadine		6 years	6 years	60 mg/day – 6 to 11 years	30 mg/day- 6 months to < 2 years
					180 mg/day ≥ 12 years	60 mg/day – 2 to 11 years
						180 mg/day ≥ 12 years
	Diphenhydramine		6 years	2 years	75 mg/day – 6 to 12 years;	37.5 mg/day – 2 to 5 years
					150 mg/day > 12 years	150 mg/day – 6 to 11 years
						300 mg/day ≥ 12 years
**Topical immunomodulators**	Pimecrolimus	Atopic Dermatitis	2 years	2 years	Twice daily, intermittent treatment 12 months	Twice daily, intermittent treatment 12 months
	Tacrolimus		2 years	2 years	0.03%- 2 to 15 years	0.03% – 2 to 15 years
					0.1% ≥ 16 years	0.1% ≥ 16 years
					(twice daily for 3 weeks then once daily, intermittent use)	(twice daily for intermittent use)

*Data obtained in the Summaries of Product Characteristics (SPC) of one European country (Portugal, *Infarmed*[47]) ^**#**^Data obtained in the FDA approved Drugs Database [48].

Comparison between an European country (regulated by EMA and the national authority) and the United States of America (regulated by FDA).

In a recently published study assessing off-label use in an allergy outpatient clinic, the most frequently prescribed drugs were nasal corticosteroid in 76% of all prescriptions, anti-histamines were used off-label in 22% [12]. T’Jong *et al.* study reported that of all respiratory drug prescriptions assessed to be used in a pediatric population, half of the patients were prescribed antihistamines and nasal corticosteroids in an off-label basis [46]. In other studies assessing systemic anti-histamines, off-label prescribing ranged from 6.5% to 43% [17,23,30,33,35]. Cetirizine [65,66], levocetirizine [62,67] and loratadine [68] have been the most investigated for long term safety in pediatric population. Despite pharmacokinetic studies have been performed in new generation anti-histamines, long-term safety studies in children are still lacking [69,70]. Indeed, due to the proven efficacy of nasal corticosteroids and anti-histamines on disease control in children by reducing disease-associated impairment and improving disease-related quality of life, more studies are needed about safety in order for physicians to perform a rational decision of the large number of options available in the market [62,69,71].

### Off-label medicines use for treating urticaria and atopic eczema in children

In a joint initiative, the European Academy of Allergology and Clinical Immunology (EAACI), the EU-funded network of excellence, the Global Allergy and Asthma European Network (GA2LEN), the European Dermatology Forum (EDF) and the World Allergy Organization (WAO) published a guideline for urticaria management [72]. In it was recommended as the first line treatment for urticaria the use of oral anti-histamines in an up-dosing step up therapy until up to 4 times the dose. These new recommendations were also advised for children, adjusting the dose accordingly to the weight [72]. Recent randomized, double-blind, placebo controlled trials in adults support the efficacy and safety of this up-dosing use, namely in cold contact urticaria [73,74]. Nevertheless, due to the absence of controlled trials in children, these changes were not updated in the SPC of the anti-histamines in the market and as stated above only a few of them were actually studied for their long term effects in children. This explains why a large portion of the off-label type of use when considering anti-histamines is due to a different dose prescription [12]. For chronic disease it is also important not only efficacy and safety, but also compliance to the treatment. Children pediatric formulations, namely under 6 years of age, are usually liquid and it is necessary to make them stable, sterile, pleasant and long lasting. Furthermore as children grow, drug doses should be adapted to weight and, to avoid dosing errors, the means to deliver accurate doses of these liquid formulations need to be available [62]. In atopic dermatitis, anti-histamines also are considered as potential benefit to reduce pruritus, and although no evidence exists to support their role in treatment they can be useful in reducing this disturbing symptom in children [69].

Accordingly to the most recently published guidelines for atopic dermatitis the main treatment is skin hydration, topical anti-inflammatory medications and antipruritic therapy [75-77]. For anti-inflammatory medication, topical glucorticosteroids or topical calcineurin inhibitors are used. For topical corticosteroids numerous substances are available, grouped by potency. Potent and very potent corticosteroids (Group III and IV) are more likely to cause systemic or local side effects (like adrenal suppression, skin atrophy or striae) than group I (mild) and II (moderate strength); therefore the first should be avoided for treatment in infants, whose higher surface area to body weight ratio and age dependent maturation of the skin barrier function leaves them vulnerable to over-dosing [75,78]. According to the FDA, use of these products are also limited by age and duration of treatment [78]. Still and specially from birth to 4 years of age, topical corticosteroids were prescribed off-label in 13% of all prescriptions, of those 58% due to high dosage use [35]. Recent guidelines recommend that for mild disease activity, a small amount of topical corticosteroids twice to thrice weekly until reaching a mean monthly dose of 15 grams (g) in infants, 30 g in children and up to 60 to 90 g in adolescents and adults [75].

Nowadays new topical anti-inflammatory alternatives include calcineurin inhibitors and fourth generation corticosteroids. This fourth generation corticosteroids, like methylprednisolone aceponate seem to have a favorable benefit-risk-ratio in this age group [79]. Regarding topical immunomodulators, calcineurin inhibitors, like tacrolimus and pimecrolimus, as they don’t cause skin atrophy, are favored for long-term management and to be used in delicate body areas, such as the eyelid region, the perioral skin, genital area, the axilla or the inguinal fold [80]. As a result of the immunosuppressant activity of these drugs there are concerns about their potential to promote skin infections and malignancies, particularly lymphomas, following long-term treatment [80]. These drugs are only approved for children with more than 2 years of age by FDA and EMA (Table 3). Due to the high prevalence of atopic dermatitis in children, which begins in over 60% of cases during the first year of life, usually affects more sensitive-skin areas and have a higher body surface/volume ratio that enhances the risk of systemic exposure to corticosteroids, it was seen an increase of use in topical calcineurin inhibitors [77,80]. Off-label use, particularly in infants in the US, reached a high prevalence of prescriptions in 2004, approximately 525,000 (14% of yearly prescriptions) for pimecrolimus and 69,000 (7%) for tacrolimus [80]. This led FDA to include a black box warning in 2005, changed to a box warning in 2006, on the labels of topical tacrolimus and pimecrolimus. Still, further discussion has occurred and even with large epidemiological data, at current time, FDA maintains that may be “a possibility of an association” [80]. However, guidelines recommend clinicians to use tacrolimus ointment, specially for eczema on the face, eyelid, and skin folds that is unresponsive to low-potency topical steroids in children older than 2 years [75,77]. Other systemic drugs for atopic dermatitis treatment also recommended off-label in children and adolescents is cyclosporine, however only reserved in the most severe and refractory to classical treatment and usually demanding specialized care [76].

### Unmet needs

According to the World Health Organization the ideal medicine for a children is “one that suits the age, physiological condition and body weight of the child taking them and is available in a flexible solid oral dosage form that can be taken whole, dissolved in a variety of liquids, or sprinkled on foods, making it easier for children to take” [81]. However this reality is far from us and still, as it was seen, drugs are not adequately studied for children.

In order to improve drug use and safety of treatment in children there is a need to increase research not only for new drugs, but also in medicines that are in market but are not adequately adjusted for children. Several worldwide regulatory efforts, namely those included in the initiative “Better Medicines for Children” allowed that a large number of new products with pediatric indications and age-appropriate pharmaceutical forms to be now authorized and made available. Furthermore, a high number of agreed pediatric investigation plans indicate that further products are appearing. However, there is an imbalance between the priorities for pediatric drug research and the need of the children. This is specially visible for respiratory and allergy treatment medicines [3,10].

Furthermore, physicians frequently encounter an inconsistency in what is proposed on the guidelines and the drugs summary of product recommendations [35]. There is an urgent need for regulations of off-label prescribing not only for medical institutions, but also for physicians [82]. In a daily basis they are confronted with questions of safety, apprehension of potential ADR, efficacy and ethical issues. As Gazarian *et al.* reported most physicians believe that off-label prescribing is adequate and they are doing it considering that the benefits outweigh the risks, but due to lack of evidence, frequently they are unaware of the true balance [13].

The overall level of unlicensed and off-label pediatric prescribing suggests the need to perform well designed clinical studies in children, for that pharmaceutical industry and academic organizations should be encouraged [37,82]. The previously implemented Pediatric Use Marketing Authorization (PUMA), which offered 8 years of data protection and 10 years of market exclusivity to any new off-patent product developed exclusively for use in the pediatric population was not successful and in the last 5 years only one was granted. Probably it did not outweight the economical risks and competition with previously implemented drugs. New measures are needed to encourage the market in a new path. Furthermore, more awareness should be enforced using population-based databases to monitor off-label prescription and by that increase awareness of the true children’s needs and interests.

## Conclusions

Off-label use in children is common and differs between countries, inpatient and outpatient settings and age. Respiratory and allergy medicines are on the top of the most prescribed off-label drugs in children, nevertheless this has not been accompanied with new research of their safety and efficacy in children, specially with those drugs already in market. In this narrative review it was recognized that a large percentage of drugs prescription in an allergist daily clinical practice are off-label. It is fundamental to increase awareness of this reality, as it is the responsibility of the clinician to balance risk-benefits of the prescription. Parents/guardians should be informed and involved in the decision in order to prevent misunderstandings, increase compliance and awareness to adverse effects in the pursuance of a good clinical outcome. There is a need for new studies with a better design to access long-term safety and efficacy of respiratory and allergy on-market drugs in children, primarily in those under two years of age. New ways should be found by the competent authorities to promote more research accordingly to the patients needs, namely on respiratory and allergy field.

## Abbreviations

ADR: Adverse drug reactions; EPR-3: Expert panel report 3: Guidelines for the diagnosis and management of asthma; EAACI: European Academy of Allergology and Clinical Immunology; EDF: European Dermatology Forum; EU: Europe; EMA: European Medicines Agency; DPI: Dry Powder Inhaler; FDA: Food and drug administration; GA2LEN: Global Allergy and Asthma European Network; GINA: Global Initiative for Asthma; ICH: International conference on harmonization; ICS: Inhaled corticosteroids; LABA: Long acting β2 agonist; MDI: Metered dose inhaler; PIP: Pediatric investigation plans; PUMA: Pediatric Use Marketing Authorization; SABA: Short acting β2 agonist; SPC: Summaries of product characteristics; TEDDY: Task-force in Europe for Drug Development for the Young; US: United States of America; WAO: World Allergy Organization.

## Competing interests

The authors declare that they have no competing interests.

## Authors’ contributions

DS, IA and MMA equally contributed to writing the manuscript. All authors have reviewed and approved the final version of manuscript.
